# Comparative effects of foliar biogenic selenium nanoparticles and selenite on soybean growth, seed quality, selenium speciation and bioaccessibility

**DOI:** 10.3389/fpls.2025.1692027

**Published:** 2025-11-07

**Authors:** Solomon Musoke Ssemalawa, Emmanuel Osei Asamoah, Muhammad Raza Farooq, Gary Bañuelos, Yuanqi Wang, Haoyuan Sun, Pincheng Rao, Yukun Guo, Youtao Chen, Xuebin Yin

**Affiliations:** 1 College of Agriculture, Anhui Science and Technology University, Chuzhou, China; 2 College of Agriculture, Shanxi Agricultural University, Taiyuan, China; 3 School of Plant Protection, Anhui Agricultural University, Hefei, China; 4 USDA Agricultural Research Service, San Joaquin Valley Agricultural Sciences Center, Parlier, CA, United States; 5 Institute of Functional Agriculture (Food) Science and Technology at Yangtze River Delta, Anhui Science and Technology University, Chuzhou, China; 6 Anhui Province Key Laboratory of Functional Agriculture and Functional Food, Anhui Science and Technology University, Chuzhou, China

**Keywords:** soybean, se biofortification, bioaccessibility, se speciation, biogenic se nano particles

## Abstract

Selenium (Se) biofortification of crops presents a sustainable strategy to address Se deficiency, which globally affects nearly one billion people. Although selenite [Se(IV)] fertilizers are commonly used for biofortification strategies, concerns over their potential toxicity in plants and low bioaccessibility have prompted interest in alternative Se sources, such as biogenic Se nanoparticles (BSeNPs). A field study was conducted to explore the effects of foliar BSeNPs and Se(IV) at 5, 10, and 20 mg L^-^¹ on soybean growth, nutritional quality, Se speciation, and bioaccessibility. Application of BSeNPs at 5 mg L^-^¹ enhanced shoot biomass (54.2%), seed protein content (62.3%), and total amino acids (76.2%) compared to both the control and corresponding Se(IV) treatments. Enhanced antioxidant enzyme responses (SOD, POD) and a decline in lipid peroxidation (MDA) were also observed with BSeNPs application, indicating enhanced stress tolerance. While Se(IV) led to higher total Se accumulation, BSeNPs promoted greater enrichment of organic Se species (SeMet, SeCys, MeSeCys). *In vitro* digestion showed that total bioaccessible Se (gastric + intestinal) ranged from 45-56% for BSeNPs versus 19.6-34% for Se(IV). In conclusion, these findings indicate that foliar BSeNPs at 5–10 mg L^-^¹ were more effective than Se(IV) for improving seed nutritional quality and Se bioaccessibility in soybean biofortification.

## 1 Introduction

Selenium (Se) serves an important function in maintaining the physiological health of both humans and animals as a crucial trace element, primarily due to its significant immunomodulatory and antioxidant properties (Sadler et al., 2024). As a constituent of over 25 selenoproteins, it plays a vital role in essential metabolic functions, notably those related to thyroid hormone synthesis and function, reproduction, and defense against oxidative stress (Wang et al., 2022). However, Se deficiency continues to pose a significant global health issue, with nearly one billion individuals affected (Wang et al., 2022). This deficiency arises primarily from a diet with less Se than the advised 55 μg per day for adults and 60 and 70 μg for pregnant women and breast feeding mothers, respectively (Winkel et al., 2012). According to Zhu et al (Zhu et al., 2017), this insufficient dietary intake is dependent on the low Se content in soils used for farming which directly influences the Se concentrations in food crops. Consequently, studies have found that both inadequate and excessive Se intake can affect human health with deficiency linked to chronic disorders such as Alzheimer’s disease (Mrština et al., 2024), hypothyroidism, cardiovascular diseases, and certain cancers (Rayman, 2012), while excess intake may lead to selenosis and related toxicity (MacFarquhar et al., 2010). However, the present work focuses on addressing Se deficiency that primarily arises from the low Se content in soils and food crops such as soybean. In combating this deficiency, Se biofortification practices have been adopted in major crops like rice, wheat, maize and various vegetables (Lyons et al., 2009; Feng et al., 2013). Inorganic forms of Se such as selenite and selenate have been predominantly used in the past for biofortification due to their high solubility and bioavailability (Zhu et al., 2017). Nanotechnology, however, is a recent emergence that has opened up vast possibilities, leading to substantial advancements in numerous domains. Nanobiotechnology, a key area within this field, focuses on the synthesis of nanoparticles using living organisms (Zohra et al., 2022) offering a promising eco-friendly and sustainable alternative to conventional supplementation methods (Bahrulolum et al., 2021). Selenium nanoparticles (SeNPs) synthesized through biological methods have attracted considerable interest due to their superior characteristics, such as enhanced stability, greater bioactivity, reduced toxicity, and improved bioavailability at optimal concentrations (Kumar and Prasad, 2021). Additionally, SeNPs have been reported to boost enzymatic activity and alleviate oxidative stress in plants (Hussein et al., 2019).

Soybean (*Glycine max*) is an economically significant legume valued for its high content of proteins, oils, and polysaccharides, as well as its contribution to soil fertility. Its exceptional protein composition makes soybean a prime candidate for Se biofortification through agronomic interventions (Lončarić et al., 2024). While numerous research has been carried out on Se biofortification in soybean using inorganic forms such as selenite and selenate (Dai et al., 2020; Silva et al., 2022), the application of BSeNPs remains largely underexplored. Inorganic Se species, that is selenite and selenate, occur as oxyanions in positive oxidation states (Wang et al., 2022) and are readily taken up by plants through sulfate or phosphate transporters. However, their high solubility and reactivity often result in oxidative stress. In contrast, BSeNPs consist of elemental Se in a zero-valent and relatively redox-stable form, which is associated with lower phytotoxicity and enhanced antioxidant responses in plants (Estevez et al., 2014). These chemical differences translate into distinct physiological outcomes. For instance, in soybean sprouts, selenite treatment produced higher malondialdehyde (MDA) levels than SeNPs, indicating greater lipid peroxidation under the ionic form (Rao et al., 2022). Similarly, in common bean, selenate treatment resulted in lower biomass production, reduced accumulation of beneficial bioactive compounds, and decreased levels of key seed metabolites compared with SeNPs (Abdelsalam et al., 2025). In *Brassica napus*, BSeNPs improved germination, boosted seedling growth, enhanced photosynthetic capacity and secondary metabolism, and conferred greater salt tolerance than Se(IV) (El-Badri et al., 2022). Likewise, in radish, BSeNPs increased yield and favored the accumulation of organic Se species in edible tissues (Huang et al., 2023). Although both conventional nano-Se and BSeNPs can enhance plant performance, we selected BSeNPs because their biological production provides sustainable synthesis and a lower-toxicity, redox-stable Se source (Xu et al., 2018) supporting our soybean biofortification objectives. Despite these promising findings, current studies on BSeNPs are predominantly limited to early developmental stages or short-term experiments on plant sprouts and seedlings. Per our review of the literature, no study has thoroughly investigated the effects of BSeNPs across the full growth cycle of soybean from vegetative stages to seed maturity nor assessed their comparative impact with inorganic Se on whole-plant physiological and nutritional processes. This study therefore aimed at evaluating the effects of foliar-applied BSeNPs and Se(IV) applied at different concentrations on soybean growth, amino acid content, protein content, nutrient uptake, Se speciation, and bioaccessibility. By addressing this gap, the research will offer new insights into the feasibility of using BSeNPs as a more potent alternative to inorganic Se fertilizers for producing high-quality, Se-enriched soybean with improved nutritional value.

## 2 Materials and methods

### 2.1 Experimental location

The experiment was conducted at Wangying Village Experimental Base in Nanqiao district, Chuzhou city, Anhui province, China (32°07’42” N and 118°24’19” E) between July and October 2024. The region experiences subtropical monsoon climate (He, 2022) characterized by four distinct seasons where mean annual temperatures fall within the range of 14°C to 22°C and has an annual precipitation of 1200 mm. Prior to conducting the field experiment, the soil was sampled and analyzed for its chemical composition and the results were as follows: pH = 6.68; organic matter = 9.08 g kg^-^¹; total nitrogen = 0.07 g kg^-^¹; total phosphorus = 0.30 g kg^-^¹; total potassium = 13.60 g kg^-^¹; available nitrogen = 0.49 g kg^-^¹; available phosphorus = 28.60 mg kg^-^¹; available potassium = 138.10 mg kg^-^¹; and selenium content = 0.19 mg kg^-^¹.

### 2.2 Experimental design and materials

**Figure 1:**
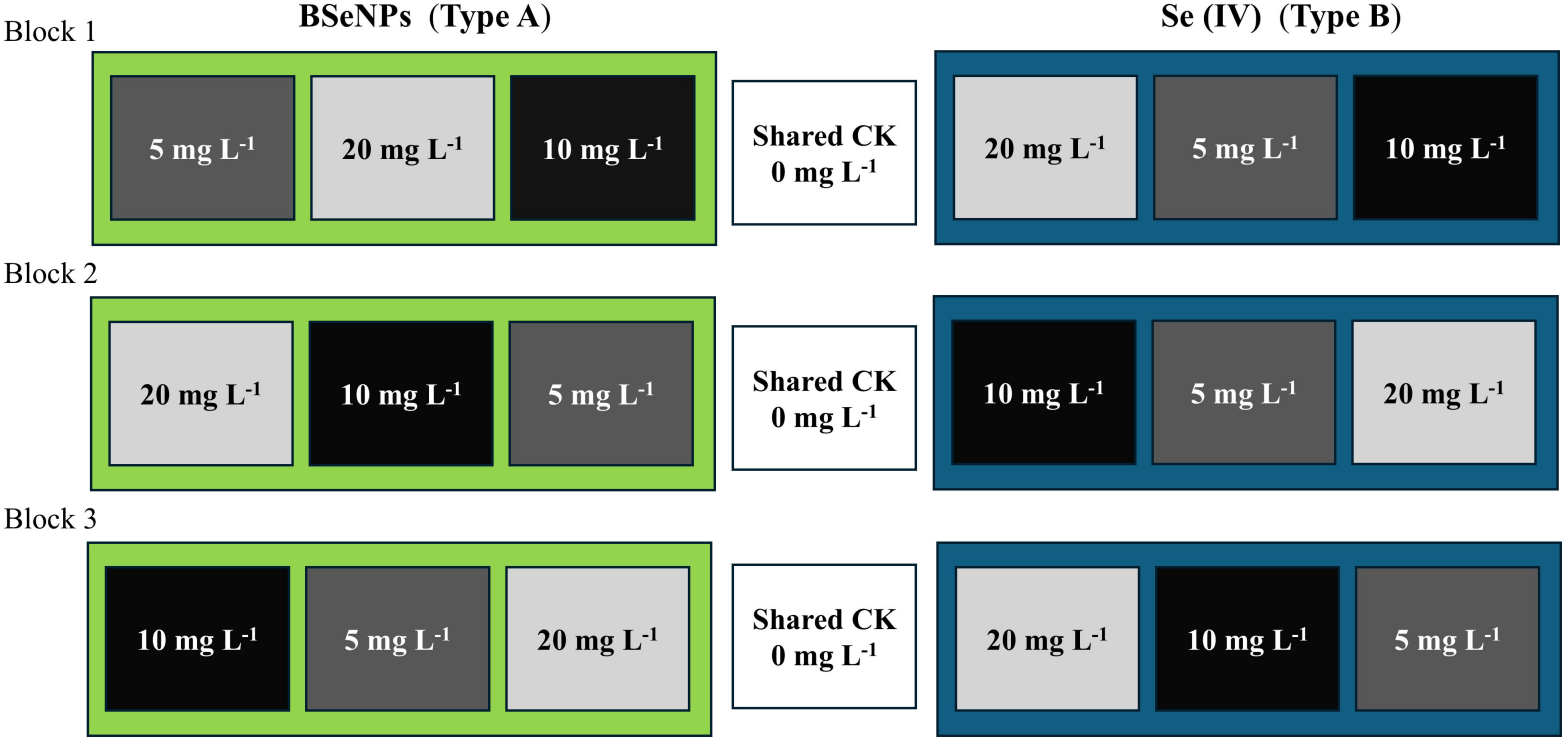
Schematic of the randomized complete block design (RCBD) with three blocks. BSeNPs (Type A) plots at 5, 10, and 20 mg L^-^¹; Se(IV) (Type B) plots at 5, 10, and 20 mg L^-^¹. A shared control (CK, 0 mg L^-^¹) was included in each block. Box order within each block indicates the randomization used in the field.

**Figure 2:**
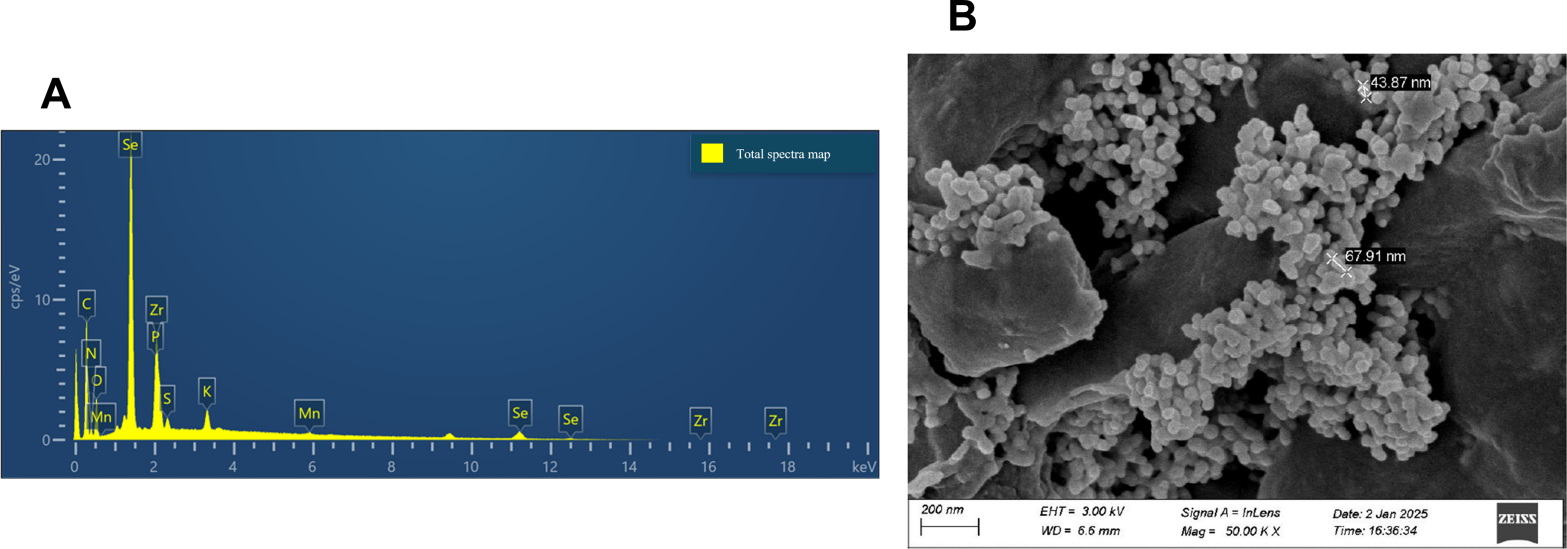
Characterization of BSeNPs; **(A)** Energy-dispersive X-ray spectroscopy (EDX), **(B)** Scanning electron microscopy (SEM).

A randomized complete block split-plot design with three blocks was used. The main plots consisted of two Se types; Se(IV) and BSeNPs assigned within each block. Within each main plot, subplots received foliar Se at rates of 5, 10, or 20 mg L^-^¹ (Wang et al., 2022), with the order randomized in every block. A single 0 mg L^-^¹ control subplot was established per block as a shared reference for both Se types and positioned between the two main plots. Each subplot measured 2 × 2m with buffer rows and alleyways maintained. In total, the field contained 21 subplots. The factor structure and the field arrangement of plots are shown in **Figure 1**. The Se(IV) used in this experiment was obtained from Shanghai Aladdin Bio-chemical Technology Co., Ltd (Shanghai, China), and the BSeNPs were produced by Jiaozuo Bai Yi’an Biological Engineering Co., Ltd. (Henan, China: product no. BYA2023081701). According to the manufacturer, the nanoparticles are produced via microbial bio-reduction using *Lactobacillus plantarum*; the detailed process is proprietary. Scanning electron microscopy (SEM) and Energy-dispersive X-ray spectroscopy (EDX) were conducted to confirm the successful synthesis and characterize the morphology and elemental composition of the BSeNPs (**Figure 2**). SEM image showed predominantly spherical particles with slight agglomeration, ranging in size from 43.9 to 68 nm. Although we did not directly profile the organic corona, the EDX detected C, N, O, P, and S in addition to Se, a pattern commonly associated with protein or polysaccharide capping layers on BSeNPs and with enhanced colloidal stability via steric effects (Xu et al., 2019; Sans-Serramitjana et al., 2023). We therefore interpret our results as the response to the supplied nanoparticles which likely include an organic surface layer. Basic fertilization was applied before sowing using a 15-15–15 ratio of N: P_2_O_5_: K_2_O fertilizer at a rate of 750 kg ha^-^¹ which was the recommended application amount (50kg mu^-^¹) by the manufacturer (Jianfeng Chemical Co., Ltd). ‘WanDou 15’ soybean seeds were sown on July 20th, with two seeds per hill at a spacing of 10 × 50 cm, resulting in approximately 160 plants per plot and a planting density of 400,000 seeds ha^-^¹. Selenium sprays were prepared per subplot by multiplying the target concentration by the fixed spray volume (1.5 L), yielding 7.5, 15, and 30 mg Se for the 5, 10, and 20 mg L^-^¹ treatments, respectively. The Carrier solution was deionized water with 0.15% v/v Tween-80 for enhanced absorption (Yang et al., 2021). Each solution was bath-sonicated for 10 mins to ensure uniform dispersion. The 0 mg L^-^¹ control used the same carrier without Se. Sprays were applied with a hand sprayer at flowering stage (55 days after sowing, DAS) until full leaf wetting was achieved, delivering exactly 1.5 L to each plot. Throughout the growing season, plants were routinely monitored, and standard agronomic practices, including pest and weed management, were implemented. Harvesting was conducted on October 18th.

### 2.3 Analytical indicators and methods

#### 2.3.1 Plant growth and yield parameters

Chlorophyll content was estimated 21 days post-treatment by measuring SPAD values on the uppermost, fully expanded leaves using a SPAD-502 chlorophyll meter. At physiological maturity (93 DAS), identified by complete pod and leaf yellowing, seed detachment from the pod membrane, and reduced seed moisture, five plants per treatment were randomly selected for agronomic measurements. Plant height was measured with a measuring tape from the stem base at soil level to the apex of the main stem. Shoots were weighed fresh immediately after harvest on a digital balance, then oven-dried at 60°C to constant mass to determine shoot dry weight. Yield parameters were defined as number of pods per plant and 1000-seed weight. Pods per plant were counted manually on each sampled plant at harvest, and values were averaged by treatment. For 1000-seed weight, clean seeds from representative subsamples were oven-dried at 60°C to constant mass and then weighed to obtain the mean 1000-seed mass per treatment.

#### 2.3.2 Enzyme activity and lipid peroxidation

At 14 days post-treatment (DPT), the uppermost fully expanded trifoliate leaves were collected from five plants per subplot, rinsed with deionized water, blotted dry, pooled to one composite sample per subplot (n = 3 per treatment), flash-frozen in liquid N_2_, and stored at −80°C. Catalase (CAT) activity was quantified spectrophotometrically by introducing enzyme extract into sodium phosphate buffer containing 0.3% H_2_O_2_ and monitoring the decline in absorbance at 240 nm; CAT activity was expressed as U g^-^¹ min^-^¹ fresh weight (FW) (Luo et al., 2019). Peroxidase (POD), superoxide dismutase (SOD), and malondialdehyde (MDA) were measured following established methods (Kong et al., 2017) (Akcin, 1974) (Luo et al., 2019),, respectively. The detailed procedures as used in this study (including slight modifications, reagent compositions, reaction conditions, and calculations) are provided in **Supplementary Methods T1-T4** respectively.

#### 2.3.3 Total se and macronutrient content

At physiological maturity, roots, shoots, and grains were sampled for total Se determination. Roots were carefully dug up and rinsed free of adhering soil, shoots were cut at the soil line, and mature grains were collected from the same plants. All tissues were oven-dried at 60°C to constant weight, ground to fine powder (≤0.5 mm), and stored in airtight tubes prior to digestion. Total Se in each tissue was quantified by ICP-MS following acid digestion as described by Carucci et al (Carucci et al., 2022), with full procedural details provided in the **Supplementary Methods T5**. Grain total nitrogen (N) was determined by the Kjeldahl method (University of Wisconsin, S) with minor modifications; the complete procedure is described in the **Supplementary Methods T6**. Phosphorus (P), and Potassium (K) concentrations were analyzed using ICP-OES according to established protocols (Cipriano et al., 2022).

#### 2.3.4 Total free amino acids and protein content

At physiological maturity, soybean grains from each subplot were oven-dried (60°C) to constant mass, finely milled (≤0.5 mm), and stored airtight until analysis. Total free amino acids were quantified by the ninhydrin method (Yemm et al., 1955) using aliquots of the milled grain extract and a glycine calibration; absorbance was read per the cited protocol. Crude protein in the grains was measured using the Bradford assay (Bradford, 1976).

#### 2.3.5 Se speciation

The analytical procedure followed that by Wang et al. (2022) with minor adaptations. Briefly, 0.5 g of soybean grain powder was hydrolyzed in 10 mL of 40 mmol L^-^¹ Tris-HCl buffer (pH 6.0) containing lipase (30 mg) and protease XIV (60 mg), assisted by ultrasonication at 37°C for 1 h, and then centrifuged at 10–000 r min^-^¹ for 30 min at 4°C. The supernatant was filtered (0.22 µm) and separated on a Hamilton PRP-X100 anion-exchange column using 40 mmol L^-^¹ diammonium hydrogen phosphate as the mobile phase (1.0 mL min^-^¹), and Se species were quantified by HPLC-ICP-MS under optimized conditions. Calibration used individual standards of selenocysteine (SeCys), methylselenocysteine (MeSeCys) and selenomethionine (SeMet) (≥99% purity) obtained from Sigma-Aldrich company and certified Se(IV) ≥97% and Se(VI) ≥98% stock solutions obtained from Tianjin Fuchen Chemical Reagent Factory and Beijing Xiya Chemical Industry Co., Ltd respectively to prepare a five-point mixed calibration (1–50 µg L^-^¹).

#### 2.3.6 Se bioaccessibility

The Physiologically Based Extraction Test (PBET) method applied in this study was adapted from the protocol established by Zhou et al. (2019) with some modifications. The simulated digestion process consisted of two sequential phases: gastric and intestinal. In the gastric phase, approximately 2.0 g of homogenized grain powder were combined with 20 mL of gastric solution in a sealed centrifuge tube. The gastric solution contained pepsin (1.25 g L^-^¹), maleic acid (0.5 g L^-^¹), citric acid (0.5 g L^-^¹), acetic acid (500 µL L^-^¹), and DL-lactic acid (420 µL L^-^¹), with the pH adjusted to 2.5 using concentrated hydrochloric acid. The mixture was incubated in a thermostatic shaking water bath at 37°C for 1 hour. After incubation, the samples were centrifuged, and the supernatant was collected and filtered to obtain the gastric digest. For the intestinal phase, the pH of the residual mixture was adjusted to 7.0 using saturated sodium bicarbonate solution. Then, 2 mL of intestinal solution containing porcine bile salts (1.5 g L^-^¹), pancreatin (0.4 g L^-^¹), and α-amylase (0.1 g L^-^¹) were added. The mixture was incubated under the same conditions (37°C) for 4 hours. Following digestion, the samples were centrifuged and filtered to obtain the intestinal digest. All extracted digests were adjusted to a final volume of 50 mL and stored at 4°C for subsequent selenium analysis. The maleic acid, acetic acid, and DL-lactic acid were sourced from Sigma-Aldrich (USA); bile salts and α-amylase from Macklin Biochemical Co., Ltd. (Shanghai, China); and other reagents from Aladdin Reagent Co., Ltd. (China). Data for Bioaccessibility (BA) was calculated using the formula below:


$$BA\%=\frac{Se\;in\;G\;or\;I}{Se\;in\;sample}\times 100$$

where Se in G and I denote the selenium levels measured during the gastric and intestinal digestion phases, respectively (mg kg^-^¹). Se in the sample indicates the selenium concentration in the original sample (mg kg^-^¹).

### 1.1 Statistical analysis

Data were organized in Microsoft Excel 2016 and reported as mean ± SD unless otherwise specified. Statistical analyses were conducted in Origin 2021 using one-way ANOVA and split-plot two-way ANOVA (Se Type as the whole-plot factor and Dose as the subplot factor) to test main effects and their interaction (Type × Dose) at α = 0.05. Full ANOVA tables are provided in **Supplementary Table S1**. Figures were prepared in GraphPad Prism 8.

## 3 Results

### 3.1 Soybean growth and yield responses

**Figure 3:**
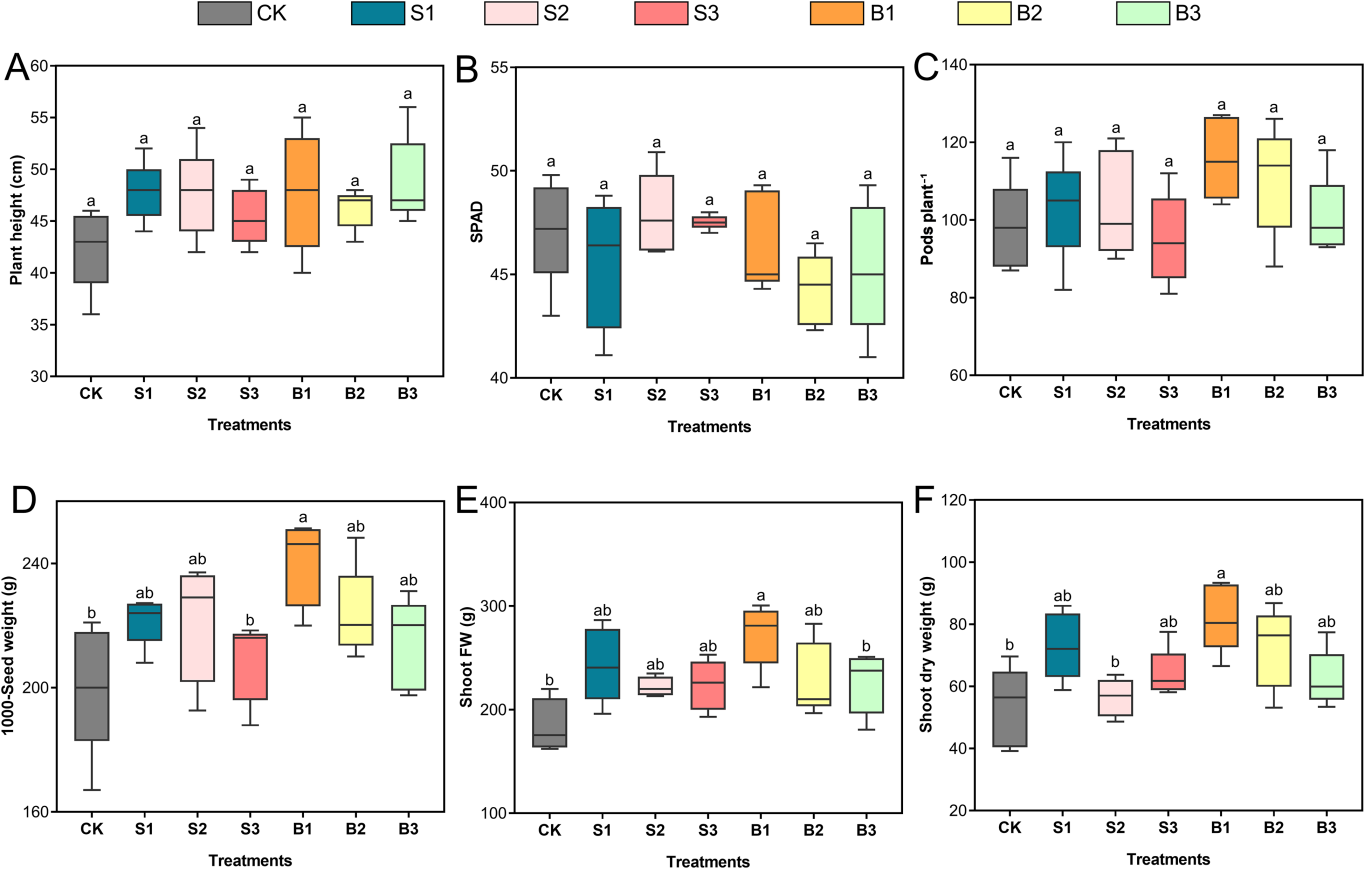
Influence of BSeNPs and Se(IV) applications on soybean growth and yield traits. **(A)** Plant height, **(B)** SPAD chlorophyll index, **(C)** Number of pods per plant, **(D)** 1000-seed weight, **(E)** Shoot fresh weight, and **(F)** Shoot dry weight. Treatments include CK (no Se), B1, B2, and B3 (BSeNPs at 5, 10, and 20 mg L^-^¹, respectively), and S1, S2, and S3 (Se(IV) at 5, 10, and 20 mg L^-^¹, respectively). Statistical analysis was conducted using one-way ANOVA followed by Tukey’s HSD test at p< 0.05. Different lowercase letters above box plots denote statistically significant differences among treatments. Data are presented as mean ± standard deviation (n = 5).

The application of various Se forms elicited differential responses in soybean growth and yield attributes, as presented in **Figure 3**. Treatments involving BSeNPs are denoted as B1, B2, and B3, while those with Se(IV) are indicated as S1, S2, and S3; the untreated control is represented as CK. Plant height exhibited modest increases across all Se-treated groups relative to the control with the most pronounced enhancements observed under B3 (15.1%) and S1 (12.7%). Nonetheless, these changes were not statistically significant (**Figure 3A**). SPAD values exhibited slight fluctuations across treatments, with the highest increase observed under S2 (1.6%) and the most notable reduction under B2 (6.1%) relative to control. Despite these variations, none of the treatments led to a statistically significant change in chlorophyll content (**Figure 3B**). The number of pods per plant increased, most notably under B1 (18.2%) and B2 (12.7%) relative to control, followed by modest gains under S1 and S2. Conversely, S3 resulted in a slight 3.1% decrease. Although numerical differences were evident, statistical analysis revealed no significant variation among treatments (**Figure 3C**). Seed weight improved across all Se treatments relative to control. The most substantial increase was observed under B1, which recorded a 19.9% enhancement. Moderate increases were also observed with B2 (11.8%), S1 (10.6%), and S2 (10.3%), suggesting a positive response to both Se forms at moderate application levels. In contrast, smaller gains were recorded under B3 (7.0%) and S3 (4.1%) (**Figure 3D**). Both Se(IV) and BSeNPs treatments enhanced shoot biomass compared to control. The most pronounced improvements were observed under B1, with shoot fresh (**Figure 3E**) and dry weights (**Figure 3F**) increasing by 48% and 54.2%, respectively. S1 also showed substantial gains, with fresh and dry weights increasing by 32.2% and 37.0%.

### 3.2 MDA content and antioxidant responses

**Figure 4:**
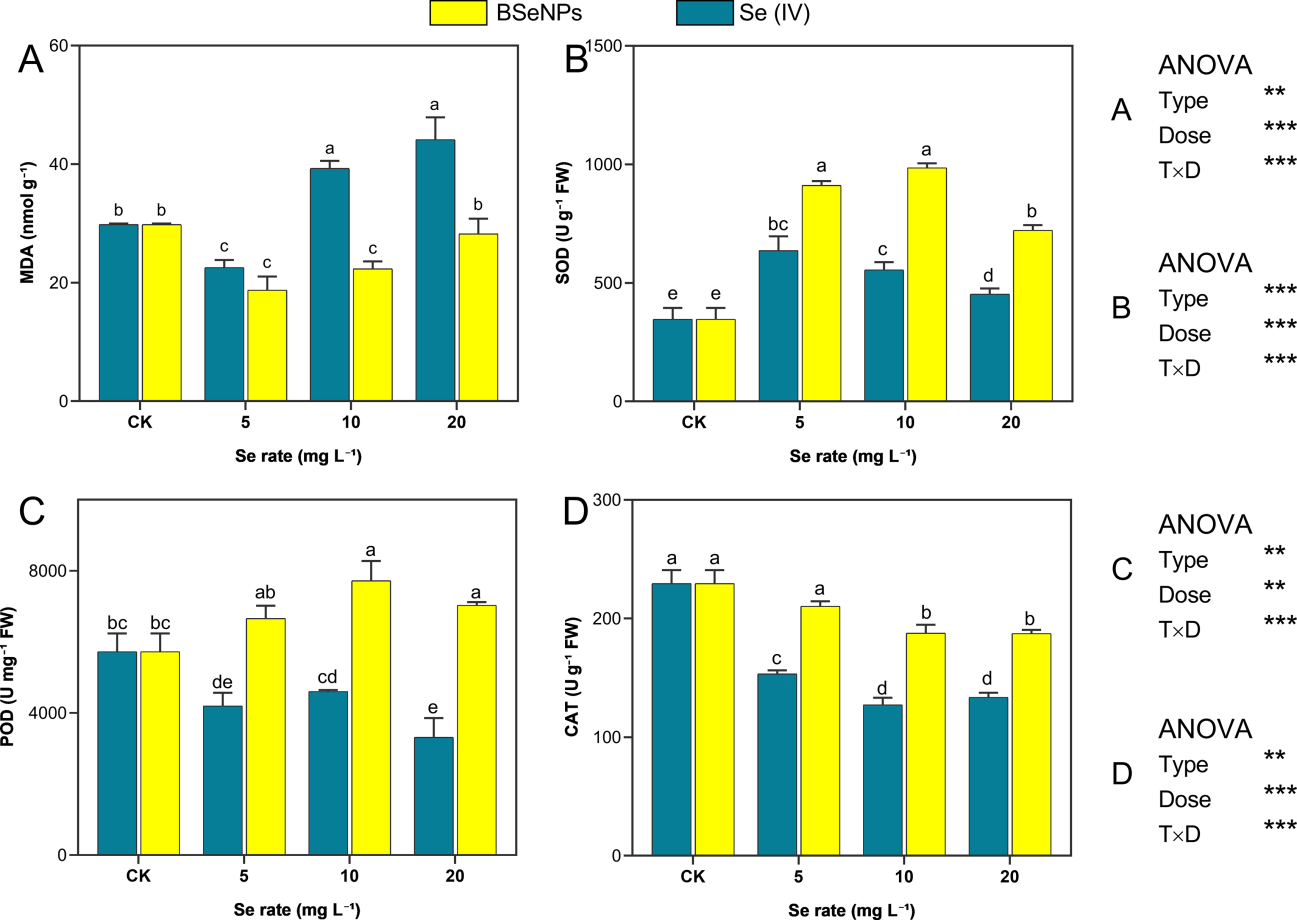
Modulation of MDA and antioxidant enzymes under BSeNPs vs Se(IV): **(A)** MDA, **(B)** SOD, **(C)** POD, **(D)** CAT. Mean ± SD (n = 3). Split-plot two-way ANOVA (Type: BSeNPs/Se(IV); Dose: 0, 5, 10, 20 mg L^-^¹). Significance for Type, Dose, and Type×Dose (ns ≥ 0.05; *< 0.05; **< 0.01; ***< 0.001), as shown in the in-panel box. Tukey’s HSD (α = 0.05); bars sharing a letter are not significantly different.

The influence of BSeNPs and Se(IV) on MDA levels, as well as antioxidant enzyme activities (SOD, POD, and CAT) are shown in **Figure 4**. At 5 mg L^-^¹, both Se forms significantly reduced MDA levels, with BSeNPs and Se(IV) achieving reductions of 45.2% and 23.6%, respectively, relative to control. However, at 10 and 20 mg L^-^¹, contrasting trends emerged with the MDA levels increasing markedly, surpassing control by 30.6% and 45.8%, respectively, in Se(IV) treatment. Conversely, BSeNPs at the same rates maintained lower MDA levels than control with reductions of 20.1% and 5.6% respectively. SOD activity increased in all treatments relative to control. Se(IV) induced increases of 95.0%, 80.2%, and 52.5% at 5, 10, and 20 mg L^-^¹, respectively, whereas BSeNPs elicited more pronounced increases of 202.1%, 215.5%, and 137.0%, respectively, at the corresponding concentrations. POD activity was elevated under BSeNPs treatment, with significant increases of 34.8% and 22.8% observed at 10 and 20 mg L^-^¹, respectively, relative to control. In contrast, Se(IV) treatment resulted in a concentration dependent decline, with the greatest reduction of 42.1% observed at 20 mg L^-^¹. CAT activity de-creased significantly under all Se treatments compared to control. In Se(IV) treated plants, activity decreased by 33.1%, 44.1%, and 42.2% at 5, 10, and 20 mg L^-^¹, respectively. Similarly, BSeNPs led to reductions of 6.5%, 17.1%, and 18.1% at the corresponding concentrations. Despite the decline, CAT activity remained significantly higher under BSeNPs than in Se(IV) at all application rates, as indicated by distinct statistical groupings. Split-plot ANOVA indicated that Se Type, Dose, and their interaction significantly affected all oxidative markers (MDA, SOD, POD, CAT); full statistics are provided in **Supplementary Table S1**.

### 3.3 Effects on macronutrients in grains

**Figure 5:**
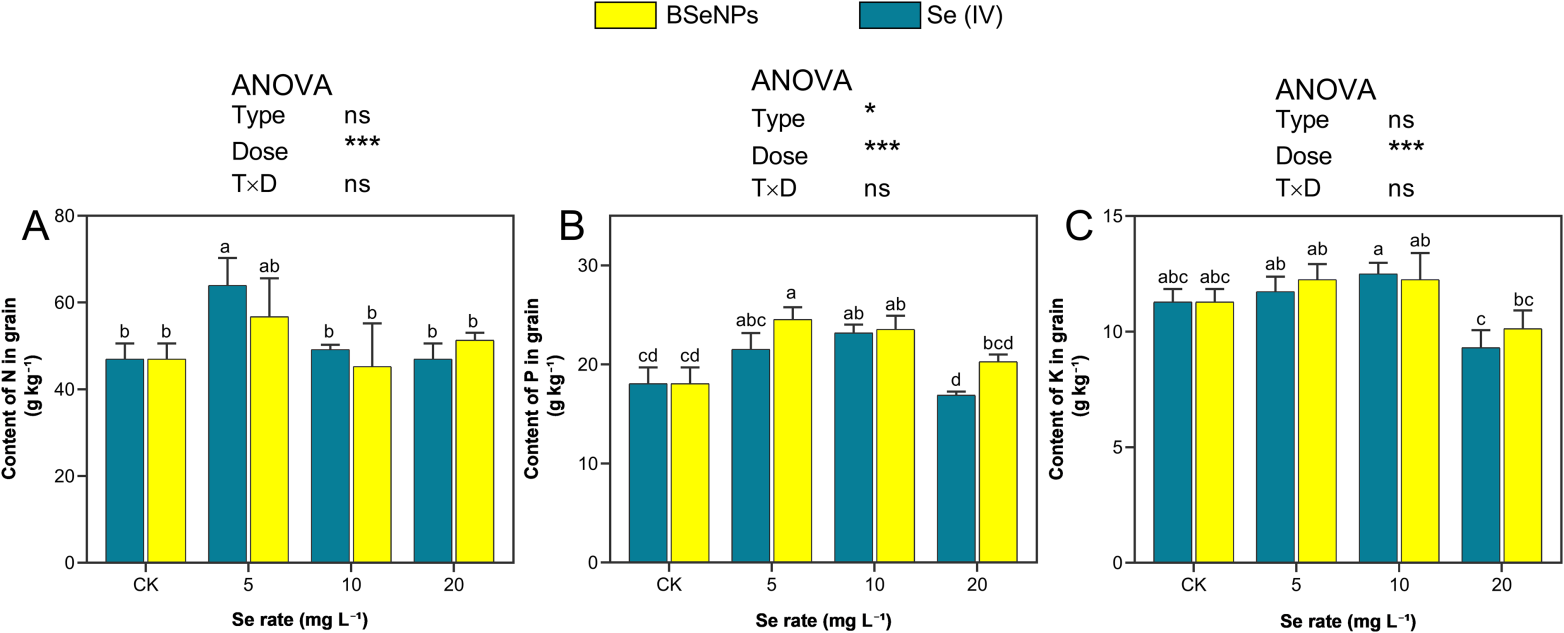
Macronutrients in soybean grain under BSeNPs vs Se(IV): **(A)** Nitrogen, **(B)** Phosphorus, **(C)** Potassium. Mean ± SD (n = 3). Split-plot two-way ANOVA (Type: BSeNPs/Se(IV); Dose: 0, 5, 10, 20 mg L^-^¹). Significance for Type, Dose, and Type×Dose (ns ≥ 0.05; *< 0.05; **< 0.01; ***< 0.001), as shown in the in-panel box. Tukey’s HSD (α = 0.05); bars sharing a letter are not significantly different.

The Se treatments significantly influenced the concentrations of macronutrients N, P, and K in soybean grains with responses varying by treatment level and Se form (**Figure 5**). Nitrogen content increased notably at 5 mg L^-^¹, with Se(IV) and BSeNPs treatments enhancing levels by 36.4% and 20.9%, respectively, in contrast with control. However, the N content at higher concentrations (10 and 20 mg L^-^¹) was not significant. Phosphorus accumulation showed a positive response following Se application at 5 and 10 mg L^-^¹, with the highest increase observed with BSeNPs at 5 mg L^-^¹ corresponding to a 22.7% increase over the control, while Se(IV) at the same rate resulted in a 19.6% increase. In contrast, P content declined at 20 mg L^-^¹, with reductions of 7.1% and 11.0% under Se(IV) and BSeNPs, respectively. Potassium accumulation exhibited a moderate but non-significant increase following Se application up to 10 mg L^-^¹, with both Se forms showing similar trends. The highest K content was observed with BSeNPs at 10 mg L^-^¹, representing a 13.8% increase compared to control. However, at 20 mg L^-^¹, both BSeNPs and Se(IV) resulted in a 10.4% and 8.6% decline in K content, respectively. Split-plot ANOVA showed that Dose significantly affected grain N and K, whereas Type and Type×Dose were not significant; for grain P, both Type and Dose were significant with a non-significant Type×Dose (see **Supplementary Table S1**).

### 3.4 Se accumulation in soybean plants

**Figure 6:**
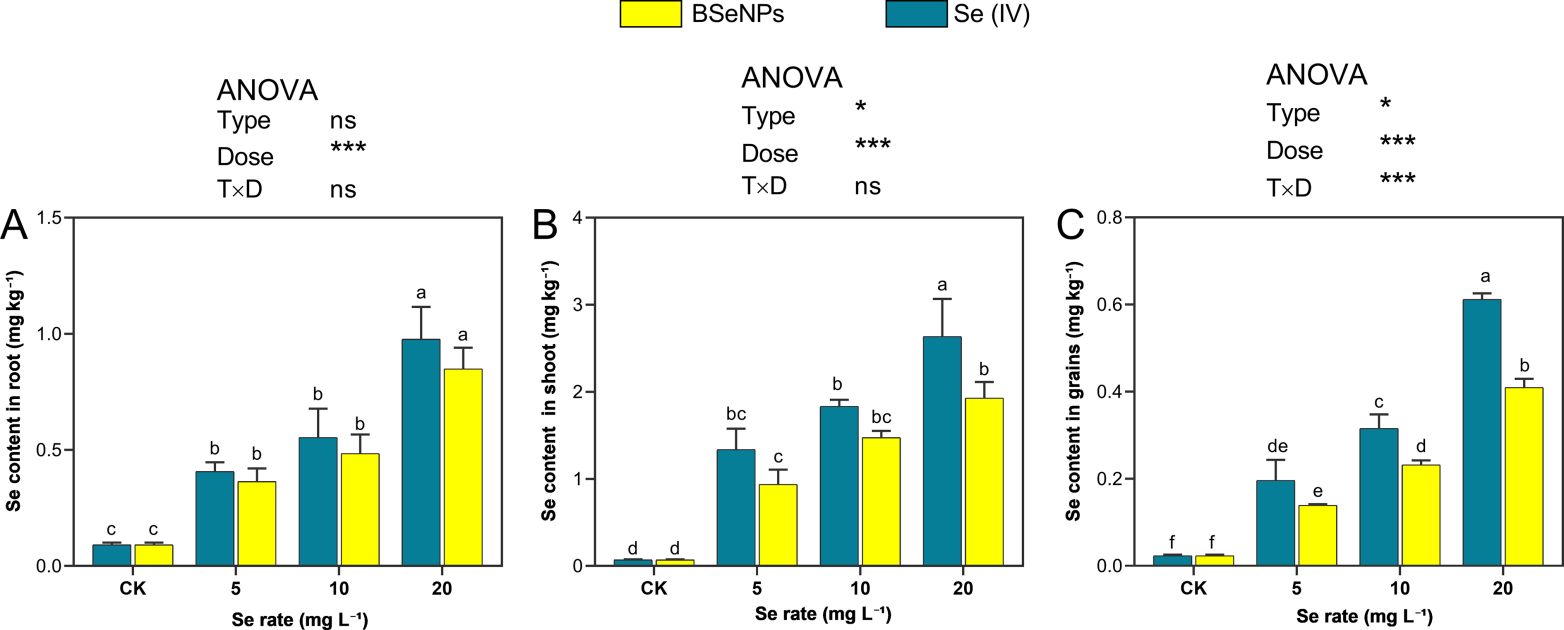
Total Se in soybean **(A)** roots, **(B)** shoots, **(C)** grains under BSeNPs vs Se(IV). Mean ± SD (n = 3). Split-plot two-way ANOVA (Type: BSeNPs/Se(IV); Dose: 0, 5, 10, 20 mg L^-^¹). Significance for Type, Dose, and Type×Dose (ns ≥ 0.05; *< 0.05; **< 0.01; ***< 0.001), as shown in the in-panel box. Tukey’s HSD (α = 0.05); bars sharing a letter are not significantly different.

Selenium distribution in soybean tissues (shoots, grains and roots) exhibited substantial differences depending on the Se form and dosage applied (**Figure 6**). Across all treatments, Se accumulation followed the consistent pattern: grain< root< shoot. In grains, Se content increased substantially at 20 mg L^-^¹ of Se(IV) and BSeNPs by 31-fold and 20.5-fold, respectively, compared to control, while lower concentrations (5 and 10 mg L^-^¹) resulted in minimal Se accumulation. The Se content in grains ranged from 0.14 to 0.41 mg kg^-^¹ with BSeNPs and 0.20 to 0.62 mg kg^-^¹ with Se(IV). In shoots, the highest Se concentrations were also observed at 20 mg L^-^¹, with Se(IV) and BSeNPs treatments yielding 37.6-fold and 27.6-fold increases, respectively. Similarly, root Se content peaked at 20 mg L^-^¹, showing 10.9-fold and 9.3-fold increases under Se(IV) and BSeNPs, respectively. Split-plot ANOVA showed that Dose significantly affected Se concentrations in all tissues (grain, shoot, root); Type was significant in grain and shoot but not in root and a Type×Dose interaction was significant only in grain. Full statistics are provided in **Supplementary Table S1**.

### 3.5 Se speciation

**Figure 7:**
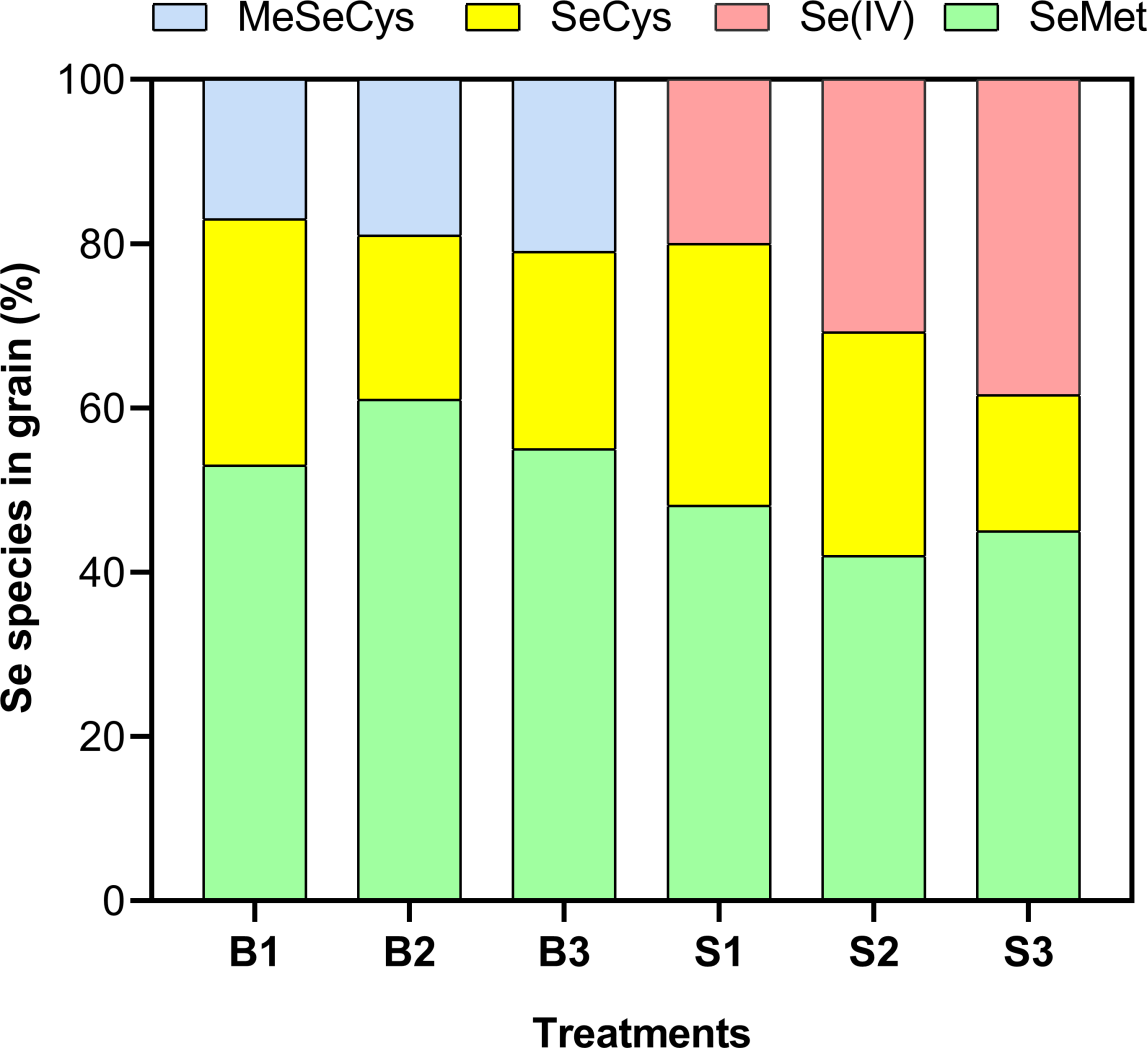
Selenium speciation in soybean grains as affected by different Se treatments. Treatments include B1, B2, and B3 (BSeNPs at 5, 10, and 20 mg L^-^¹, respectively), and S1, S2, and S3 (Se(IV) at 5, 10, and 20 mg L^-^¹, respectively). SeCys (selenocysteine), MeSeCys (methylselenocysteine), SeMet (selenomethionine) and Se(IV) (selenite).

Characterization of various chemical forms of Se revealed that organic Se species dominated across all treatments, irrespective of the Se form applied (**Figure 7**). Among the detected species, selenomethionine (SeMet) was the most abundant, accounting for 53% – 61% and 42% – 48% of total Se in plants treated with BSeNPs and Se(IV), respectively. Notably, Se(IV) was only detected in Se(IV) treated plants, with its proportion increasing significantly at higher application rates, ranging from 20% to 38.4%. The proportion of selenocysteine (SeCys) varied across treatments. In plants treated with BSeNPs, SeCys accounted for 30% of total Se species at 5 mg L^-^¹ but decreased to 20% and 24% at 10 mg L^-^¹ and 20 mg L^-^¹, respectively. Similarly, in Se(IV) treated plants, SeCys was 31.9% at 5 mg L^-^¹ but decreased to 27.2% and 16.6% at 10 mg L^-^¹ and 20 mg L^-^¹, respectively. Additionally, methylselenocysteine (MeSeCys) was exclusively present in BSeNPs treated plants, comprising 17–21% of the total Se in grains.

### 3.6 Seed protein and amino acids composition

**Figure 8:**
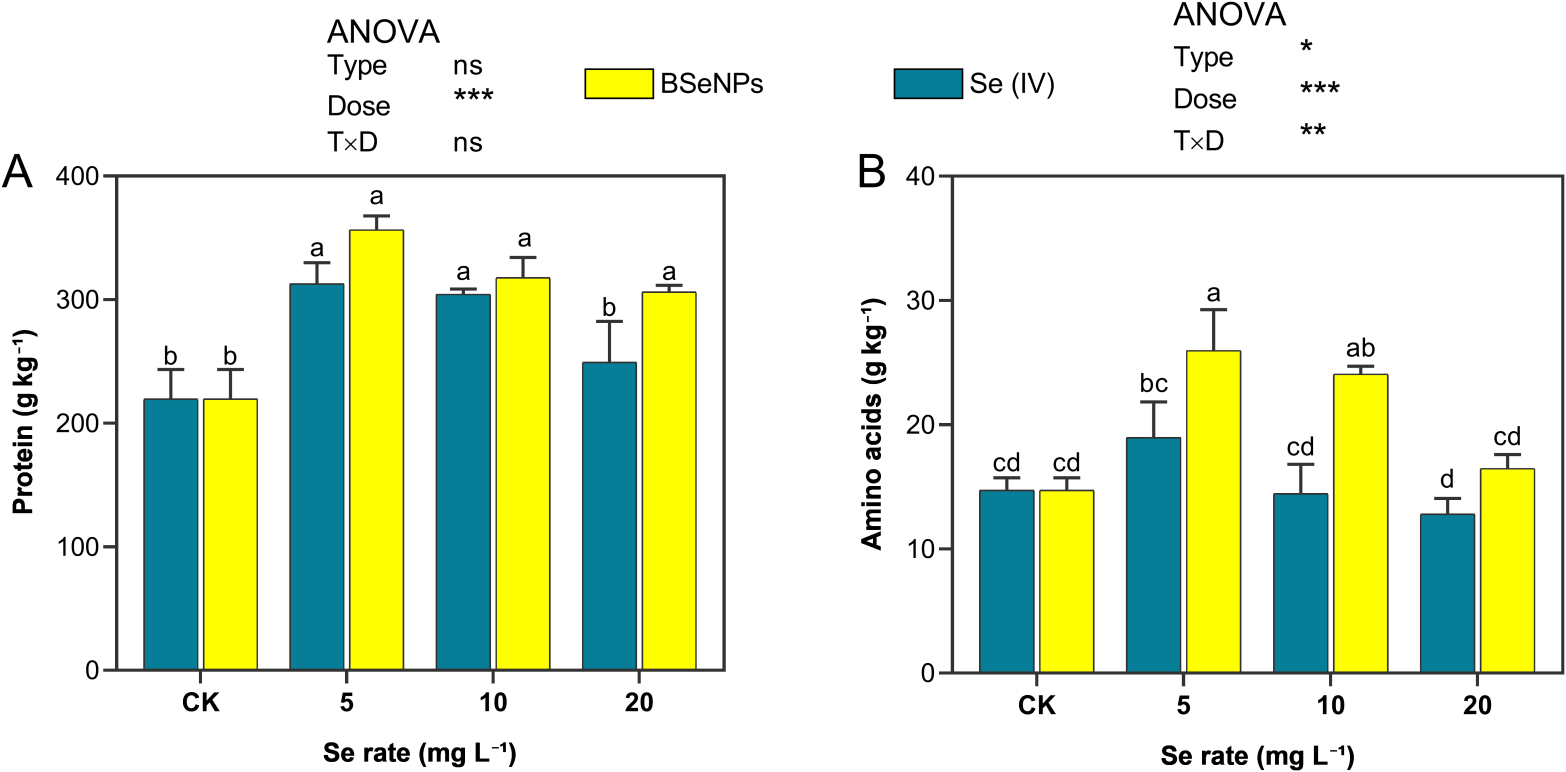
Soybean grain **(A)** protein and **(B)** total free amino acids under BSeNPs vs Se(IV). Mean ± SD (n = 3). Split-plot two-way ANOVA (Type: BSeNPs/Se(IV); Dose: 0, 5, 10, 20 mg L^-^¹). Significance for Type, Dose, and Type×Dose (ns ≥ 0.05; *< 0.05; **< 0.01; ***< 0.001), as shown in the in-panel box. Tukey’s HSD (α = 0.05); bars sharing a letter are not significantly different.

Soybean seed protein content notably increased due to the Se treatments with both BSeNPs and Se(IV) in contrast with control (**Figure 8**). In plants treated with 5, 10, and 20 mg L^-^¹ of BSeNPs, an increase of 62.3%, 44.6%, and 39.4% in seed protein was observed respectively. In contrast, Se(IV) treatment resulted in lower protein increments of 42.4%, 38.5%, and 13.5% at the same application rates. Similarly, Se treatments positively influenced the amino acid content of soybean seeds, with BSeNPs demonstrating superior effectiveness. At 5 mg L^-^¹ and 10 mg L^-^¹, BSeNPs significantly increased amino acid levels by 76.2% and 67.8%, respectively, compared to control. In contrast, Se(IV) treatments resulted in more moderate improvements, with the highest increase of 28.8% at 5 mg L^-^¹, although it was not statistically significant. Split-plot ANOVA showed that Dose significantly affected both total amino acids and protein; a Type×Dose interaction was significant for amino acids only, while Type main effects were not significant for either trait (see **Supplementary Table S1**).

### 3.7 Se bioaccessibility

The bioaccessibility of Se in soybean varied significantly between BSeNPs and Se(IV) treatments, as shown in **Table 1**. Overall, BSeNPs treatment led to a higher proportion of bioaccessible Se compared to Se(IV). During the gastric stage, Se bioaccessibility ranged from 13.3% to 17.7% in BSeNPs-treated soybean, while Se(IV) treated samples exhibited lower values, ranging from 9.37% to 11.5%. A more pronounced difference was observed in the intestinal phase, where bioaccessible Se in BSeNPs increased to 31.4%- 38.3%, compared to 10.2%-22.5% in the Se(IV) treatments. Notably, at 10 mg L^-^¹ of BSeNPs, the total bioaccessible Se increased by 24.4% and 13.8% compared to the 5 mg L^-^¹ and 20 mg L^-^¹ treatments, respectively. Conversely, in the Se(IV) treatments, increasing the concentration to 10 mg L^-^¹ and 20 mg L^-^¹ led to a 17.6% and 42.4% reduction in total bioaccessible Se, respectively.

**Table 1:** Se bioaccessibility after *in vitro* gastric and intestinal digestion.

Treatment (mg L^-1^)		Bioaccessibility (%)	
		Gastric	Intestinal
Control	0	–	–
Se(IV)	5	11.5 ± 0.33^a^	22.5 ± 1.56^a^
	10	8.32 ± 0.57^b^	19.7 ± 0.81^a^
	20	9.37 ± 0.85^a^	10.2 ± 0.50^b^
BSeNPs	5	13.6 ± 1.28^c^	31.4 ± 1.54^c^
	10	17.7 ± 0.69^d^	38.3 ± 1.16^d^
	20	13.3 ± 1.00^c^	35.9 ± 2.52^d^

Bioaccessibility (%), reported as mean ± SD (n = 3). For each digestion phase (gastric or intestinal), means followed by different lowercase letters differ significantly among treatments (one-way ANOVA, Tukey’s HSD, p< 0.05).

## 4 Discussion

### 4.1 Se treatments on growth and yield parameters

Although Se often shows a hormetic response, stimulating growth at low doses and inhibiting it at higher doses (Schiavon and Pilon-Smits, 2017), we observed no statistically significant effects of Se on plant height, SPAD, or pod number relative to the control. This likely reflects our application timing: Se treatments were applied at flowering stage (55 DAS), when vegetative growth naturally slows as assimilates are redirected to reproductive sinks. Consequently, inputs that can enhance vegetative traits at earlier stages may have limited impact at this stage. Consistent with this interpretation, prior work reports stronger responses to foliar Se at vegetative stages and reduced or absent effects when applied at flowering or pod filling (Moloi and Khoza, 2022). Nevertheless, shoot biomass values were numerically higher in Se-treated plants, with the largest directional increases in the 5 mg L^-^¹ BSeNPs and Se(IV) treatments (fresh weight 48% and 32.2% above control; dry weight 54.2% and 37%, respectively). While these differences were not significant and should be interpreted cautiously, their direction is consistent with known Se actions at low dose moderation of ROS balance and protection of photosynthetic machinery, and, in legumes, support of N metabolism which can subtly improve biomass even when point estimates do not reach significance. Notably, the BSeNPs trend exceeding Se(IV) mirrors reports of greater bioavailability and lower toxicity of BSeNPs in rape seed (El-Badri et al., 2022), however, our data do not permit firm conclusions and should be viewed as hypothesis-generating. Collectively, these findings indicate that under a flowering-stage application, Se did not alter primary architectural traits, but small, non-significant biomass gains particularly with BSeNPs are biologically plausible and warrant targeted testing at earlier growth stages or with increased replication.

### 4.2 MDA and antioxidant responses

MDA is a recognized biomarker for lipid peroxidation in plant cell membranes and serves as an important indicator of oxidative stress (Morales and Munné-Bosch, 2019). In this study, soybean plants treated with BSeNPs at 5 and 10 mg L^-^¹ exhibited significantly lower MDA levels compared to control, indicating a reduction in lipid peroxidation and enhanced oxidative stress tolerance. Previous reports have similarly highlighted the antioxidant potential of SeNPs in alleviating stress-induced peroxidation (Rao et al., 2022). Treatment with Se(IV) at 5 mg L^-^¹ also reduced MDA content, suggesting a protective effect at minimal concentrations. We, however, observed that MDA levels increased at higher Se(IV) concentrations, implying the onset of oxidative stress likely due to Se toxicity and a buildup of reactive oxygen species (ROS). This concentration-dependent response is consistent with earlier findings that excessive Se can cause oxidative damage rather than provide protective effects (Cheng H. et al., 2024; Liu et al., 2024). Supporting this concept, Feng et al. (2013) reported that Se at optimal doses effectively reduces MDA accumulation across various plant species, further emphasizing its dose-dependent physiological role. Key antioxidant enzymes like SOD, POD, and CAT are pivotal in detoxifying ROS and mitigating oxidative damage in plants (Huang et al., 2018). In this study, BSeNPs- treated plants exhibited increased activities of SOD, POD, and CAT, suggesting a more robust antioxidant defense system compared to Se(IV) treated and control plants. Similar enhancements in SOD and CAT activity have been reported in tomato plants following SeNPs treatment (Ishtiaq et al., 2023). Moreover, Samynathan et al. (2023) observed improved antioxidant capacity and growth performance in groundnut plants after foliar application of nano Se (40 mg L^-^¹), highlighting the potential of nano-Se formulations in stress mitigation. In addition to their antioxidant effects, SeNPs have been reported to upregulate genes associated with antioxidant defense, as demonstrated in strawberry plants (Huang et al., 2018). Furthermore, SeNPs can stimulate secondary metabolism, leading to the accumulation of stress-related phytochemicals such as phenolic compounds, thereby enhancing plant stress tolerance (Neysanian et al., 2020). This response can be a potential area for further and future investigation on BSeNPs.

### 4.3 Macro nutrients

In this study, an interaction between Se application and the accumulation of macronutrients (N, P, and K) in grains was observed, with effects varying based on the form and concentration of Se. At 5 mg L^-^¹, both Se(IV) and BSeNPs enhanced N content, a result consistent with previous findings in legumes and rice where low levels of Se improved N uptake and assimilation (Lei et al., 2022; Silva et al., 2023). However, increasing Se concentrations beyond this level did not yield any significant difference compared to control. Zhu et al. (2017) also observed a similar trend where higher Se conditions reduced N accumulation in various plant organs of *Codonopsis lanceolata*. This observation at higher Se concentrations may be attributed to Se’s ability to disrupt N assimilation pathways, particularly by interfering with the uptake of micronutrients like molybdenum, a key cofactor for nitrate reductase and thereby limiting the synthesis of nitrogenous compounds (Schiavon et al., 2017). P and K exhibited a comparable dose-dependent pattern, with applications at 5 and 10mg L^-^¹ performing better than control, while the highest concentrations (20mg L^-^¹) led to a reduction. BSeNPs consistently outperformed Se(IV) in promoting P and K uptake, possibly due to their influence on root physiology and rhizosphere interactions. Specifically, Se nanoparticles may stimulate root exudation and microbial activity, facilitating greater nutrient solubilization and absorption (Cheng B. et al., 2024). These findings are in agreement with reports demonstrating improved P and K accumulation following nano Se and Se(IV) treatments (Cipriano et al., 2022; Selim et al., 2022). However, the observed decline in P and K content at higher Se levels may stem from Se-induced physiological stress or antagonistic interactions that impair nutrient transport and membrane function. Excess Se may disrupt ion balance or hinder ATPase activities involved in active transport, thereby limiting P and K uptake under elevated Se exposure (Hasanuzzaman et al., 2020; Schiavon et al., 2020).

### 4.4 Se content

The application of Se notably increased Se concentration in soybean plants proportional to the level of application, indicating that both Se(IV) and BSeNPs were effectively absorbed. The accumulation pattern observed across plant parts followed this order: grain< root< shoot. Notably, Se(IV) treatments consistently resulted in higher total Se accumulation in roots, shoots and grains compared to BSeNPs, suggesting that Se(VI) was more readily absorbed and translocated in the plant system. This trend aligns with previous findings that Se(VI), due to its water solubility and transport via phosphate or sulfate transporters, tends to accumulate faster and in greater amounts than nanoparticulate forms (Li et al., 2020; Rao et al., 2022). Hu et al. (2018) also reported a significantly slower influx of BSeNPs into wheat roots compared to Se(IV), supporting the idea that nanoparticle uptake kinetics differ substantially from their ionic counterparts. Their study also revealed that the absorption efficiency of BSeNPs is influenced by particle size, with nanoparticles smaller than 50 nm exhibiting improved uptake compared to larger particles a finding that was in agreement with several others (Bano et al., 2021; Cheng B. et al., 2024; Madlala et al., 2024). Additionally, the physical limitations imposed by the size of plant cell wall pores play a crucial role, as only nanoparticles or their aggregates with diameters smaller than these pores can effectively penetrate and reach the plasma membrane (Moore, 2006; Dietz and Herth, 2011). The comparatively lower Se content observed in BSeNPs treated plants in our study may therefore be attributed to differences in particle size, uptake kinetics or the plant physiology. Nevertheless, further research is warranted to elucidate the specific mechanisms governing the absorption, transport, and translocation of BSeNPs in soybean. Despite lower total Se accumulation, several studies have indicated that BSeNPs may outperform Se(IV) in promoting plant growth, enhancing stress tolerance, and improving biochemical quality parameters (Li et al., 2020; El-Badri et al., 2022). Moreover, BSeNPs have been found to pose lower toxicity than Se(IV) (Zhang et al., 2023), which is consistent with some of the observations made in our study.

### 4.5 Se speciation

The effectiveness of Se in soybeans for human health depends on both its concentration and its bioavailable forms. In plants, Se exists in both organic forms, such as SeMet, SeCys and MeSeCys and inorganic forms, including selenite and selenate. Organic Se species are generally considered more bioavailable and bioactive, while exhibiting lower toxicity in plants compared to their inorganic counterparts (Tangjaidee et al., 2023). In our study, plants treated with BSeNPs accumulated Se in organic forms, with SeMet (53–61%) and SeCys (20–30%) as the major species, while MeSeCys (17–21%) was present in lower proportions. This distribution is consistent with the findings of Lu (Lu et al., 2018), who reported that Se-enriched wheat primarily contained SeMet, SeCys and MeSeCys. Chan et al. (2010) further highlighted that legumes, including beans, are particularly efficient in synthesizing SeMet and SeCys, suggesting that soybeans have a strong capacity to convert inorganic Se into bioavailable organic forms. This process, however, remains unclear. A key finding in our study is that BSeNPs resulted in a significantly higher proportion of organic Se species compared to Se(IV) treated plants. Although Se(IV) can also be metabolized into organic Se forms, a previous study (Pyrzyńska, 1996) has shown that plants exposed to Se(IV) may often accumulate higher levels of selenite, selenate and elemental Se in plants. In contrast, BSeNPs, due to their microbial origin, are coated with biological capping agents, e.g., proteins, polysaccharides, phenols, amines, and alcohols (Jain et al., 2015), which are likely to facilitate Se uptake and transformation by influencing enzyme-mediated pathways (Hu et al., 2018), leading to enhanced conversion of inorganic Se into organic forms. In this study, bond-level assays (e.g., FTIR, high-resolution XPS) were not performed to directly resolve the organic corona, so we interpret responses as those to the as-received material in comparison with prior studies; future work should include such analyses to substantiate capping composition and function. The increase in organic Se forms coupled with reduced inorganic species suggests improved nutritional quality of the biofortified grain, as organic Se compounds are generally more bioavailable and nutritionally desirable for human consumption (Freire Moreira et al., 2024).

### 4.6 Total free amino acids and protein content

Total free amino acid content in soybean significantly increased with BSeNPs at 5 and 10 mg L^-^¹, outperforming all Se(IV) treatments (**Figure 8**). A previous study on common bean (Abdelsalam et al., 2025), reported similar findings, where application of SeNPs enhanced amino acid levels more effectively than with Se(VI) treatments. Furthermore, Huang et al. (2024) also observed that SeNPs applied at the lowest rate of 6 mg L^-^¹ resulted in the highest increase in amino acid content in soybean sprouts, while higher concentrations led to a decrease in amino acid content, a trend that mirrors our results. This decrease at higher SeNPs concentrations may occur because at elevated levels, more SeNPs can be externally bound to plant tissues rather than being effectively integrated into amino acid metabolism, resulting in an increase in total Se content but with a decreasing amino acid content (Huang et al., 2024). Our results indicated a positive relationship between Se application and soybean seed protein content compared to control, with BSeNPs generally resulting in higher protein levels than Se(IV). A similar observation was reported in a study (Xia et al., 2020), where organic Se enrichment in wheat grains was directly associated with increased protein content. This observation suggests that the higher proportion of organic Se species in BSeNPs-treated soybean grains may have contributed to the observed increase in protein levels. However, further research is needed to confirm this relationship and to elucidate the underlying mechanisms involved. Additional studies also support the role of Se in promoting protein synthesis in plants (Mostofa et al., 2017; Xia et al., 2020; Sindireva et al., 2023). However, the favorable influence of Se on protein content appears to be dose dependent, as protein levels in soybean seeds decreased at the highest application rates. This trend aligns with findings in rice by Mostofa et al (Mostofa et al., 2017), indicating that excessive Se may impair protein biosynthesis. Conversely, Rao et al. (2022) reported an opposite pattern, where low concentrations of SeNPs or Se(IV) led to low protein content, while higher doses promoted protein accumulation. Such discrepancies may stem from differences in experimental conditions, crop species, Se forms, and application timing. Nonetheless, it is well documented that an overabundance of applied and accumulated Se may exert toxic effects on plants, resulting in metabolic imbalances that inhibit amino acid and protein synthesis (Gouveia et al., 2020; Somagattu et al., 2024).

### 4.7 Se bioaccessibility

Numerous studies have evaluated Se bioaccessibility in a range of cereals, legumes, and leafy green vegetables like rice (Jaiswal et al., 2012), wheat (Khanam and Platel, 2016), lettuce (do Nascimento da Silva et al., 2017) and radish (Hu et al., 2021) through *in vitro* methods. Currently, research examining Se bioaccessibility in soybean is limited. Some studies (Huang et al., 2022; Xiong et al., 2025) investigated Se bioaccessibility in soybean using polyphenols and peptides extracted from soybean sprouts, however, they did not compare different forms of Se. Our findings showed that BSeNPs and Se(IV) at different rates influenced total bioaccessible Se in soybean grains ranging from 45% to 56% and 19.6% to 34% in the two forms respectively. These results are consistent with the findings in soybean sprouts (43.4%), Lettuce (33.3%) and rice (40.4%) (Gong et al., 2018; Farooq et al., 2024). The distinction between the bioaccessibility of BSeNPs and Se(IV) denotes the significance of organic Se compounds in determining Se bioaccessibility. The BSeNPs were dominantly incorporated into organic Se species (**Figure 7**). This transformation plays a key role in enhancing Se solubility and absorption during digestion, leading to higher bioaccessibility compared to Se(IV) treated soybean. This observation aligns with previous findings (Muleya et al., 2021), which reported enhanced bioaccessibility of organic Se compounds, attributed to their structural compatibility with biological systems. A mechanistic explanation for this trend was provided in (Zeng et al., 2023), where Se bioaccessibility during gastric and intestinal phases showed a strong correlation with organic Se species particularly SeMet as the principal contributor alongside SeCys, and MeSeCys (r = 0.95–0.97), whereas inorganic forms like selenite and selenate exhibited weaker correlations (r = 0.26–0.28). Furthermore, the higher bioaccessibility of organic Se species can be attributed to their stability during digestion and their ability to be directly absorbed in the intestine without requiring additional transformation (Lavu et al., 2016; Zeng et al., 2023). Interestingly, across Se(IV) treatments, it was observed that as the total Se content increased, bioaccessibility tended to decrease. This observation suggests that at higher Se(IV) concentrations, certain chemical factors may have limited its absorption efficiency. Excess Se has been shown to induce oxidative stress and cytotoxic effects during digestion which may inhibit digestive enzyme activity, disrupting protein and lipid digestion, that are essential for the proper assimilation of Se-bound compounds (Chen et al., 2022). Verma et al. (2012) also noted that during digestion in the gastrointestinal tract, Se in inorganic form may recombine with other components in the digesta forming insoluble complexes that are later excreted, lowering its absorption, whereas the organic forms may be actively absorbed by means of peptide or amino acid uptake routes.

### 4.8 Pearson’s correlation matrix

**Figure 9:**
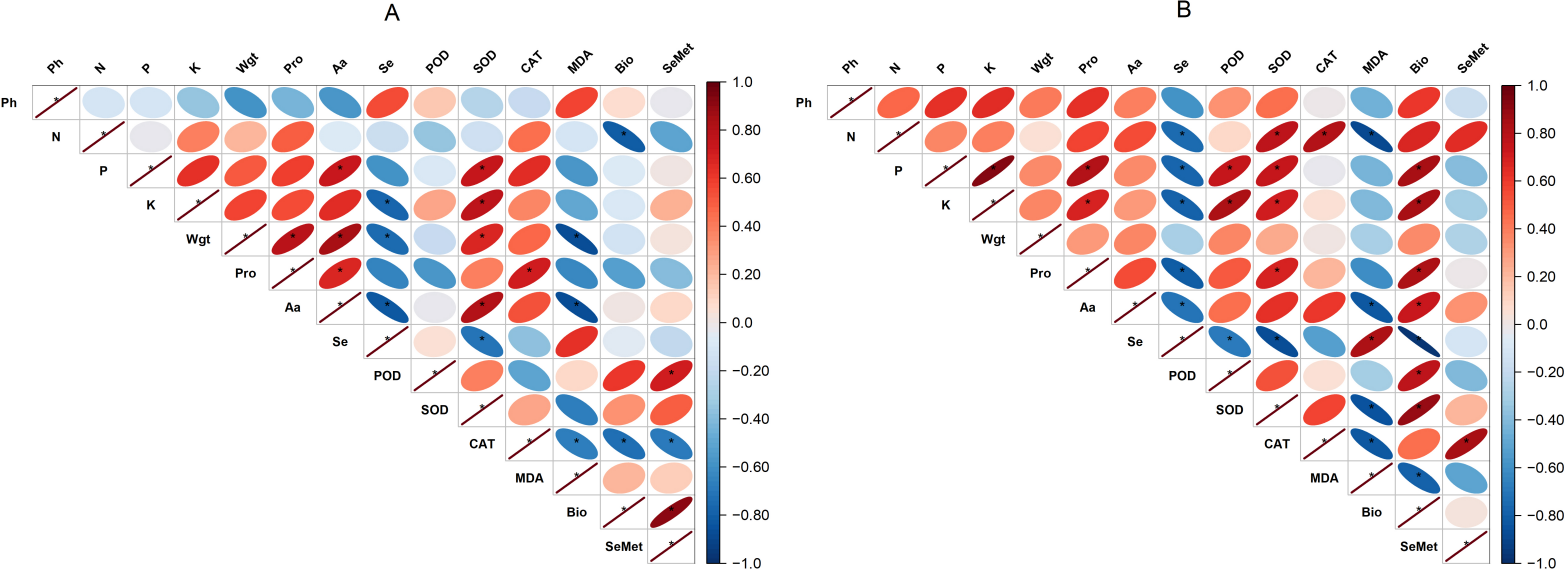
Pearson’s correlation matrices showing the relationships among soybean variables under treatments with BSeNPs **(A)** and Se(IV) **(B)**. Ph, Plant height; Wgt, Seed weight; Pro, Proteins; Aa, Amino acids; Bio, Bioaccessibility. An asterisk (*) indicates a significant positive correlation (p< 0.05).

The correlation between the variables assessed in the analysis is presented in **Figure 9**. With BSeNPs treatment, Se content showed a positive association with plant height (R² = 30%). SeMet exhibited stronger relationships, being highly correlated with bioaccessibility (R² = 83%) and positively associated with antioxidant enzymes SOD (R² = 25%) and POD (R² = 52%). In contrast, with Se(IV) treatment, Se content displayed negative correlations with other variables. For SeMet in Se(IV)-treated plants, significant positive correlations were observed with CAT activity (R² = 71%) and nitrogen concentration (R² = 44%), whereas its association with bioaccessibility was negative.

### 4.9 Metabolic transformation mechanism of BSeNPs and Se(IV)

**Figure 10:**
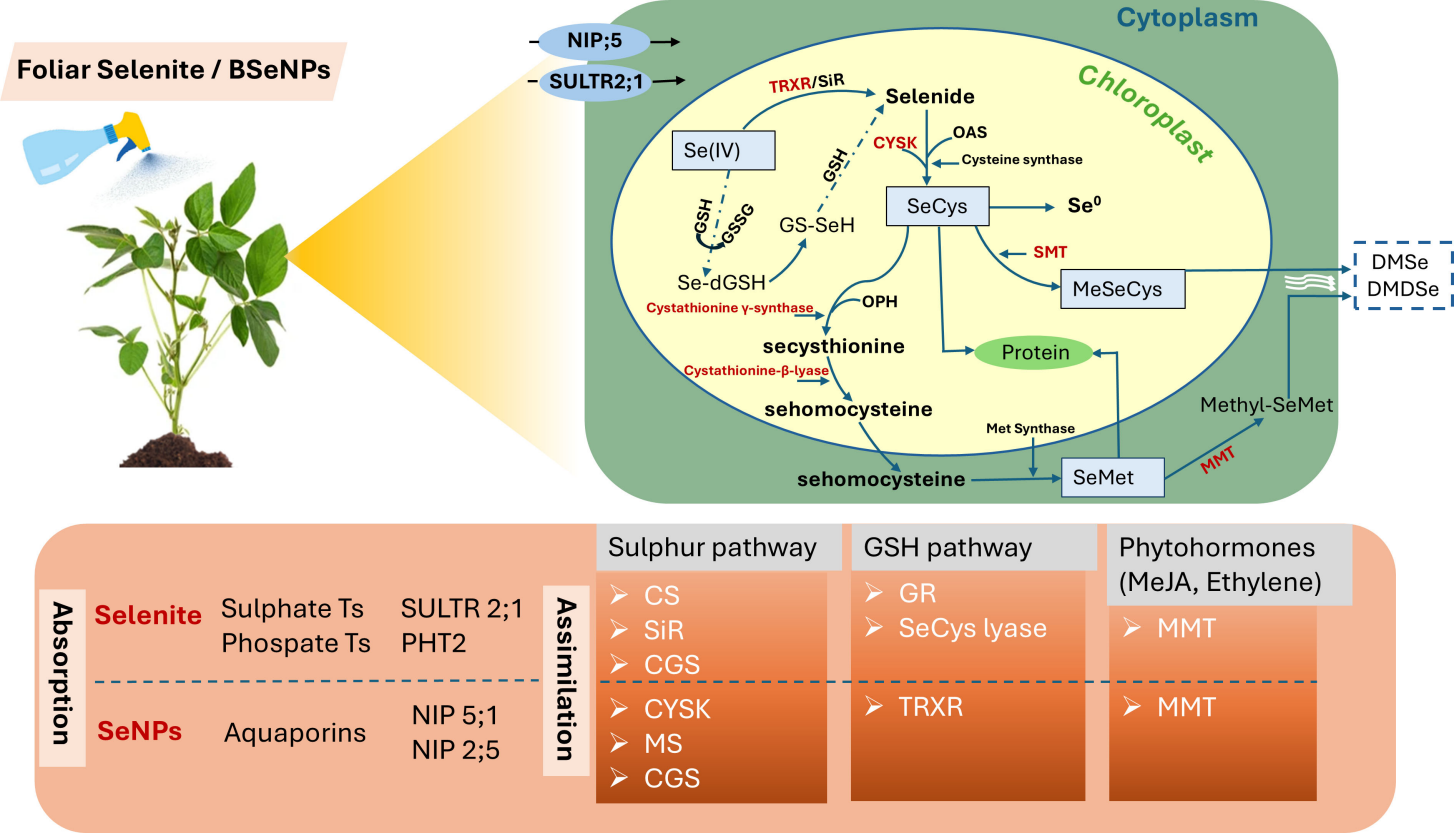
**A** conceptual illustration of Se uptake and metabolism in soybean. Selenium intake transporters involved in leaves for Se(IV) and BSeNPs are SULTR2;1, PHT2 and NIP;5 respectively. Enzymes highlighted in red represent potential regulatory activity by Adenosine 5’-phosphosulfate (AS). Enzyme annotation: TRXR (thioredoxin reductase), CS (cysteine synthase), SiR (Sulphite reductase), CYSK (cysteine synthase), GSH (glutathione), GSSG (glutathione disulfide), OPH (O-phosphohomoserine), CGS (cystathionine gamma-synthase), SMT (selenocysteine methyltransferase), MMT (methionine S- methyltransferase), MS (methionine synthase), GR (glutathione reductase).

Foliar Se application deposits nutrient-containing droplets on leaves, allowing Se to enter through cuticular pores via passive diffusion along a concentration gradient, then to the mesophyll cells through specific membrane transporters. Leaves primarily absorb Se in its inorganic forms, which are subsequently transformed into organic forms within the plant. Notably, Se(IV) and SeNPs are predominantly taken up via sulphate transporters and aquaporins respectively (Cheng et al., 2025). Upon entering the plant cells, Se(IV) undergoes a reduction to selenide (Se^2−^), facilitated either enzymatically by sulfite reductase or non-enzymatically through interactions with reduced glutathione (**Figure 10**). Se^2−^ then combines with O-acetylserine (OAS) to use cysteine synthase to form selenocysteine (SeCys) (Kurmanbayeva et al., 2022). Further metabolic pathways lead to the synthesis of elemental Se, methyl-selenocysteine (Me-SeCys), and selenomethionine (SeMet), a key precursor to selenoproteins (Lanza and Dos Reis, 2021). SeMet itself can undergo methylation by methionine methyltransferase to yield methyl-SeMet, which may be further converted into dimethylselenide (DMSe), a volatile Se compound (Pilon-Smits et al., 2009). The ratio of organic and inorganic Se species in different plant parts are largely determined by internal metabolic activities. In this study, it was found that BSeNPs were effectively converted into organic Se compounds in soybean seeds. Se(IV) also underwent transformation into organic forms, however, residual inorganic Se specifically as selenite was detected, indicating potential differences in metabolic processing. These variations may vary depending on the presence of metabolic co-factors like reduced glutathione and the expression of key enzymes. For example, limited glutathione availability can constrain the conversion of selenite to selenide, thereby affecting overall Se metabolism (Lanza and Dos Reis, 2021). Previous studies have reported that excessive Se(IV) exposure can deplete glutathione levels, hence disrupting the reduction of inorganic Se into organic forms (Kaur and Sharma, 2018; de Souza Cardoso et al., 2023) which can have potentially elevated inorganic Se in Se(IV)-treated plants. In contrast, BSeNPs, which are naturally stabilized by organic or protein-based coatings (Hu et al., 2021), tend to induce less oxidative stress and thereby improve plant metabolism and Se assimilation efficiency (Samynathan et al., 2023). Moreover, transcriptomic analyses in soybean reveal that SeNPs treatment enhances the expression of genes such as CYSK, involved in SeCys synthesis, and SMT, associated with MeSeCys production. CGS and METE may be downregulated at high SeNP concentrations also favoring MeSeCys accumulation (Xiong et al., 2023). Nonetheless, further investigation is necessary to elucidate the mechanisms and biochemical pathways governing the transformation of BSeNPs into organic Se species in plants.

## 5 Conclusion

This study demonstrates that treatment of soybean with foliar BSeNPs at 5 and 10mg L^-^¹ offers a dose-efficient biofortification strategy compared to Se(IV). BSeNPs at these levels significantly improved soybean shoot biomass, seed protein content, and total amino acid levels, while simultaneously alleviating oxidative damage, as indicated by decreased MDA levels and increased antioxidant enzyme activities (SOD, POD). Notably, BSeNPs promoted a higher proportion of organic Se compounds (SeMet, SeCys and MeSeCys) in soybean grains, forms that were not only more beneficial for plant nutrition but also exhibited superior bioaccessibility. Although Se(IV) resulted in higher total Se accumulation, BSeNPs may offer enhanced nutritional advantages in biofortified soybean.

## Data Availability

The original contributions presented in the study are included in the article/**Supplementary Material**. Further inquiries can be directed to the corresponding author/s.
