# Supplementary material for: Comparative effects of foliar biogenic selenium nanoparticles and selenite on soybean growth, seed quality, selenium speciation and bioaccessibility
Source: Front Plant Sci. 2025 Nov 7;16:1692027. doi: 10.3389/fpls.2025.1692027 (PMC12634606; doi:10.3389/fpls.2025.1692027)
Supplement: Supplementary file 1 [file DataSheet1.pdf]

frontiers

Supplementary Material

Supplementary Table S1. Two-way split-plot ANOVA summary for effects of Se type, foliar dose, and their interaction on physiological and nutritional traits in soybean.

Trait	Source	SS	DF	MS	F-value	p-value	Sig
MDA	Type	502.7011	1	502.7011	126.9085	0.007788	**
MDA	Dose	749.6528	3	249.8843	61.6932	1.45E-07	***
MDA	Type × dose	327.7215	3	109.2405	26.97007	1.28E-05	***
MDA	Residual	48.60522	12	4.050435			
SOD	Type	356178.5	1	356178.5	1357.794	0.000736	***
SOD	Dose	733980.3	3	244660.1	183.1694	2.77E-10	***
SOD	Type × dose	143807.5	3	47935.84	35.88807	2.85E-06	***
SOD	Residual	16028.45	12	1335.704			
POD	Type	32435870	1	32435870	103.0928	0.009561	**
POD	Dose	3241036	3	1080345	6.198203	0.008694	**
POD	Type × dose	11981770	3	3993923	22.91411	2.95E-05	***
POD	Residual	2091597	12	174299.8			
CAT	Type	11006.67	1	11006.67	198.6318	0.004997	**
CAT	Dose	19938.09	3	6646.029	160.4514	6.01E-10	***
CAT	Type × dose	3700.845	3	1233.615	29.78249	7.66E-06	***
CAT	Residual	497.0498	12	41.42081			

N	Type	17.4251	1	17.4251	1.923963	0.299778	ns
N	Dose	728.1069	3	242.7023	12.97095	0.000449	***
N	Type × dose	113.5425	3	37.84752	2.022717	0.164493	ns
N	Residual	224.5347	12	18.71123			
P	Type	17.0522	1	17.0522	47.19462	0.020538	*
P	Dose	143.411	3	47.80367	25.35966	1.76E-05	***
P	Type × dose	13.87428	3	4.62476	2.453417	0.113498	ns
P	Residual	22.6203	12	1.885028			
K	Type	0.448267	1	0.448267	1.252564	0.379435	ns
K	Dose	24.82003	3	8.273344	18.41374	8.72E-05	***
K	Type × dose	1.054233	3	0.351411	0.782125	0.526438	ns
K	Residual	5.391633	12	0.449303			
Se root	Type	0.021811	1	0.021811	3.154866	0.217685	ns
Se root	Dose	2.092198	3	0.697399	95.9214	1.19E-08	***
Se root	Type × dose	0.013131	3	0.004377	0.601998	0.626029	ns
Se root	Residual	0.087246	12	0.007271			
Se shoot	Type	0.803004	1	0.803004	24.27293	0.038815	*
Se shoot	Dose	15.77709	3	5.259031	120.216	3.22E-09	***
Se shoot	Type × dose	0.375137	3	0.125046	2.858417	0.081422	ns
Se shoot	Residual	0.524958	12	0.043747			

Se grain	Type	0.044033	1	0.044033	34.31228	0.027929	*
Se grain	Dose	0.759571	3	0.25319	594.6537	2.6E-13	***
Se grain	Type × dose	0.032666	3	0.010889	25.57333	1.69E-05	***
Se grain	Residual	0.005109	12	0.000426			
Protein	Type	4881.769	1	4881.769	14.2504	0.063557	ns
Protein	Dose	44766.4	3	14922.13	35.34303	3.09E-06	***
Protein	Type × dose	3107.915	3	1035.972	2.453696	0.113471	ns
Protein	Residual	5066.504	12	422.2087			
AA	Type	154.1787	1	154.1787	62.71974	0.015572	*
AA	Dose	259.7783	3	86.59276	23.89004	2.39E-05	***
AA	Type × dose	78.12798	3	26.04266	7.184898	0.005106	**
AA	Residual	43.49567	12	3.624639			

Supplementary Table S1. Two-way split-plot ANOVA for the effects of Se Type (whole-plot; BSeNPs or Se(IV)), Dose (subplot; 0, 5, 10, 20 mg L^{-1}), and their interaction (Type×Dose) on enzyme activity and lipid peroxidation (CAT, POD, SOD, MDA), macro-nutrients (N, P, K), Se content (root, shoot, grain), protein content, and total free amino acids (AA). Reported statistics: SS - Sum of Squares, DF - Degrees of Freedom, MS - Mean Square, F-value, and p-value, Sig - Significance: * $p<0.05$, ** $p<0.01$, *** $p<0.001$, **ns** = not significant.

Supplementary Methods

T1

CAT activity

CAT was assayed at 25 °C by monitoring H_2O_2 decomposition at 240 nm. Fresh leaf tissue (0.2 g) was homogenized on ice in 2 mL ice-cold 50 mM sodium phosphate buffer (pH 7.0) containing 1% (w/v) polyvinylpyrrolidone and 1 mM ethylenediaminetetraacetic acid (EDTA). The homogenate was centrifuged (12,000 g, 15 min, 4 °C) and the supernatant used immediately. The reaction (1-cm quartz cuvette) contained 2.90 mL 50 mM phosphate buffer (pH 7.0), 0.05 mL freshly prepared 0.3% (w/v) H_2O_2, and 0.05 mL enzyme extract; a reagent blank lacked enzyme. The linear decrease in A_{240} over 1–2 min was recorded, and activity was calculated using $\varepsilon(H_2O_2, 240\ nm) = 39.4\ M^{-1}\ cm^{-1}$ and expressed as U g^{-1} FW.

T2

POD activity

POD was quantified following the guaiacol–H_2O_2 method. Fresh tissue (0.2 g) was chilled, blotted dry, and homogenized on ice in 2 mL of ice-cold 50 mM potassium phosphate buffer (pH 7.0) containing 1% (w/v) polyvinylpyrrolidone and 1 mM ethylenediaminetetraacetic acid (EDTA). The homogenate was clarified by centrifugation (12,000 g, 15 min, 4 °C) and the supernatant (enzyme extract) was kept on ice and used immediately. Assays were run at 25 °C in a 1-cm quartz cuvette by monitoring the increase in absorbance at 470 nm due to guaiacol oxidation. Unless otherwise noted, the 1.00 mL reaction contained 50 mM potassium phosphate (pH 6.5), 10 mM guaiacol, 10 mM H_2O_2, and 50 µL enzyme extract; reactions were initiated by adding H_2O_2, mixed quickly, and the linear ΔA_{470} min^{-1} was recorded over 60–120 s against a reagent blank lacking enzyme. Activity was calculated as µmol tetraguaiacol formed per minute using the molar absorptivity $\varepsilon_{470} = 26.6\ mM^{-1}\ cm^{-1}$ (pathlength = 1 cm), and reported as U mg^{-1} FW.

T3

SOD activity

SOD was measured by the NBT–riboflavin photochemical inhibition assay at 25 °C. Fresh leaf tissue (0.2 g) was ground in liquid N_2 and extracted on ice with 100 mM potassium phosphate (pH 7.8) containing 1% (w/v) PVPP and 0.1 mM EDTA (3 mL per 0.2 g). The homogenate was centrifuged (12,000 ×g, 15 min, 4 °C) and the supernatant used immediately. Reactions (1.00 mL; 1-cm cuvette) contained 50 mM phosphate (pH 7.8), 13 mM L-methionine, 75 µM NBT, 0.1 mM EDTA, 2 µM riboflavin, and 50 µL extract; illumination with cool-white fluorescent light for 10 min generated superoxide, with “light, no-enzyme” and dark blanks in parallel. Absorbance at 560 nm was read immediately; SOD activity was calculated from the percent inhibition of NBT reduction vs. the light control (blank-corrected). One unit was defined as 50% inhibition under these conditions, and activities were reported as U g^{-1} FW.

T4

MDA activity

MDA was measured by reacting with thiobarbituric acid (TBA). Fresh tissue (0.2 g) was ground in liquid N_2 and homogenized on ice with 5% (w/v) trichloroacetic acid (TCA). Homogenates were centrifuged (12,000 rpm, 15 min, 4 °C) and 0.5 mL supernatant was combined with 1.0 mL 0.5% (w/v) thiobarbituric acid prepared in TCA. Mixtures were heated at 95 °C for 30 min, cooled to room temperature, and briefly recentrifuged (7,500 rpm, 5 min) to clarify. Absorbance of the supernatant was read at 532 and 600 nm against a 5% TCA blank in 1-cm cuvettes. MDA was calculated as MDA (nmol g^{-1} FW) = $\{[A_{532} - A_{600}] / (\varepsilon \times W)\} \times 10^6$, with $\varepsilon = 155\ mM^{-1}\ cm^{-1}$ and W the fresh mass (g) extracted.

T5

Se content

Soybean shoot, root, and grain samples were finely milled to powder, and 0.5 g of each was weighed into a 10 mL Xpress microwave-digestion vessel. 5.0 mL concentrated HNO_3 and 1.0 mL 30% (v/v) H_2O_2 were added, the vessel was loosely capped for 30 min at room temperature to allow pre-reaction, and samples were then digested in a microwave system (800 W) using a stepped program of 130 °C for 5 min (15 °C min^{-1} ramp) followed by 160 °C for 15 min (10 °C min^{-1} ramp). After cooling, digests were quantitatively diluted to 25.0 mL with ultrapure water, mixed, and filtered through 0.22 µm nylon. Selenium was determined by ICP-MS (Agilent 7900 ICP-MS, Agilent Technologies Inc., Santa Clara, USA) with continuous acquisition of ^{77}Se, ^{78}Se, and ^{80}Se under typical operating conditions (RF power 1550 W, sample depth 8 mm, plasma gas 15 L min^{-1}, auxiliary gas 0.9 L min^{-1}, standard conical nebulizer 1.0 L min^{-1}). Quantification used external calibration on ^{78}Se prepared in the same acid matrix as samples, with reagent blanks, a certified reference material, and spike-recovery checks included for quality control. Results were reported as mg kg^{-1} dry weight.

T6

Nitrogen content

Total Kjeldahl nitrogen (TKN) was determined on dried, finely ground soybean grain. 0.5 g of homogenized powder was placed in a 100 mL Kjeldahl digestion tube and moistened with 1 mL water. Concentrated H_2SO_4 (5 mL) was added, the tube was swirled, and 30% H_2O_2 was introduced in two 2 mL portions; the tube was covered with a small funnel and heated on a digestion block until the initial vigorous reaction subsided and the mixture darkened. After a brief cooling, 10 drops of H_2O_2 were added and the digest was reheated to a gentle boil for 5 min; this boil step was repeated with further small H_2O_2 aliquots (total 6 mL) until the solution became clear and dense white fumes were observed, then heating continued for 5 min to remove excess H_2O_2. After cooling, the digest was quantitatively transferred to a 100 mL volumetric flask, diluted to volume with ultrapure water, mixed, and filtered; a reagent blank was processed in parallel. For distillation, ammoniacal nitrogen liberated by alkalization was steam-distilled into 30 mL boric acid acceptor (10 g L^{-1} with mixed methyl-red) on an automated Kjeldahl unit configured with 40 mL distilled water and 20 mL NaOH (400 g L^{-1}); a 20 mL aliquot of digest was used per run, with a blank distilled first. The collected borate solution was titrated with 0.01 mol L^{-1} H_2SO_4 to the indicator endpoint, and total nitrogen was calculated from titrant consumption (blank-corrected) using the standard Kjeldahl expression that accounts for titrant normality, digest aliquot and final volume, and sample mass. Results were expressed on a dry-weight basis and reported as g kg^{-1}; values were converted from %N by multiplying by 10.
